# The Chemical Constituents and Pharmacological Actions of *Cordyceps sinensis*

**DOI:** 10.1155/2015/575063

**Published:** 2015-04-16

**Authors:** Yi Liu, Jihui Wang, Wei Wang, Hanyue Zhang, Xuelan Zhang, Chunchao Han

**Affiliations:** ^1^School of Pharmacy, Shandong University of Traditional Chinese Medicine, Jinan 250355, China; ^2^Teaching Experiment Center, Shandong University of Traditional Chinese Medicine, Jinan 250355, China; ^3^College of Acumox and Tuina, Shandong University of Traditional Chinese Medicine, Jinan 250355, China

## Abstract

*Cordyceps sinensis*, also called DongChongXiaCao (winter worm, summer grass) in Chinese, is becoming increasingly popular and important in the public and scientific communities. This study summarizes the chemical constituents and their corresponding pharmacological actions of *Cordyceps sinensis*. Many bioactive components of *Cordyceps sinensis* have been extracted including nucleoside, polysaccharide, sterol, protein, amino acid, and polypeptide. In addition, these constituents' corresponding pharmacological actions were also shown in the study such as anti-inflammatory, antioxidant, antitumour, antiapoptosis, and immunomodulatory actions. Therefore can use different effects of *C. sinensis* against different diseases and provide reference for the study of *Cordyceps sinensis* in the future.

## 1. Introduction

The genus* Cordyceps* is an important kind of medicinal fungi belonging to the Ascomycota, Pyrenomycetes, Hypocreales, and Clavicipitaceae [1–5].* Cordyceps* are specific macrofungi because of their characteristic parasitic habit on larvae and pupae of insects. As a pleomorphic fungus distributed worldwide,* Cordyceps* is particularly abundant in tropical forests and humid temperate [3–5]. Within the genus* Cordyceps*, over 400 species have been described so far [4, 5], of which* Cordyceps sinensis*, also called as “winter worm, summer grass,” is recognized as the most famous tonic herb in traditional Chinese medicine (TCM) for centuries.

*Cordyceps sinensis* is an abundant resource in nature with various biological activity and has been used extensively as a tonic and health supplement for subhealth patients especially seniors in China and other Asian countries. Till now, numerous bioactive constituents have been extracted such as cordycepin, polysaccharides, ergosterol, mannitol, and adenosine [6, 7]. Meanwhile, various pharmacological actions of these chemical constituents have been reported, including antitumour effect, hepatoprotective and inflammatory effects, and antioxidant, nephroprotective, and antiapoptotic properties [8–14]. To sum up, the effect of* C. sinensis* may be caused by a single active ingredient or by the combined action of many active agents that existed in the extract.

Research is necessary to get an overview about the genus* Cordyceps sinensis* because of the increasing interest both for medicine and mycology [15, 16]. Therefore, our study has reviewed the chemical constituents and their corresponding pharmacological actions of the* Cordyceps sinensis* for its significant role in the development of new drugs and therapeutics for various diseases. Moreover, realizing the pharmacological action of the monomer composition could strengthen the drug efficacy through extracting a single ingredient in* Cordyceps sinensis*. Therefore, it is necessary to review the development on the research of* C. sinensis*.

## 2. Chemical Constituents and Their Corresponding Pharmacological Actions of* Cordyceps sinensis*

### 2.1. Nucleosides

Nucleosides, a major active component of* C. sinensis*, are used as a valuable chemical marker for quality control of* Cordyceps* [1]. Besides, nucleosides play an important role in the drug development of cancer and infectious diseases, and nucleosides and their derivatives have been widely used in anticancer and antiviral therapies. Since 3′-deoxyadenosine, namely cordycepin, was isolated from cultured* Cordyceps militaris*, nucleosides in* Cordyceps* have become a focus [1]. In succession, more than ten nucleosides and their related compounds, including adenine, adenosine, inosine, cytidine, cytosine, guanine, uridine, thymidine, uracil, hypoxanthine, and guanosine, have been isolated from* Cordyceps sinensis*. Almost all of the nucleotides and nucleosides in* C. sinensis* can be transformed reciprocally [17]. Furthermore, many scholars began to study its pharmacological effects and had a lot of achievements [18, 19]. An UPLC method for fast simultaneous determination of several nucleosides was developed this year and was also applied for the determination of the analytes in cultured* Cordyceps sinensis* [19, 20]. Then, a series of researches about nucleosides was carried out quickly. For example, nucleosides can adjust and control the human body physiological activities through purinergic and/or pyrimidine receptors [17]. Therefore, determination of nucleosides and their related compounds is extremely important for the pharmacological study and quality control of* C. sinensis* and its products.

#### 2.1.1. Cordycepin

**Figure 1:**
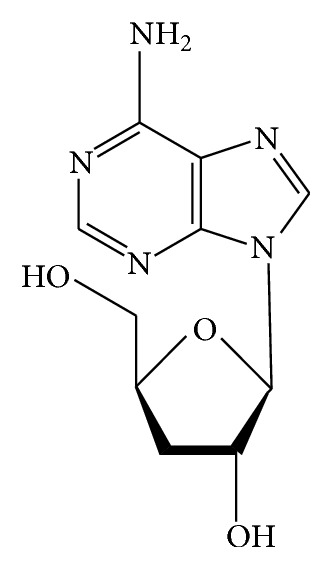
Chemical structure of cordycepin.

Early in 1950, cordycepin was first isolated from* C. militaris* and its structural formula was confirmed as 3′-deoxyadenosine but it is only found in natural* C. sinensis* with very low content and cannot be detected in the cultured ones [17]. Cordycepin is the most considerable adenosine analogue from some* Cordyceps* [21], which is a derivative of the nucleoside adenosine differing from the latter by the absence of oxygen in the 3′ position of its ribose entity (Figure 1). Cordycepin was separated with a mixture of acetonitrile and water (5 : 95, v/v) at a flow rate of 1.0 mL/min, which is the commonly used method to extract the composition [22].

Cordycepin is a category of compounds that exhibits significant therapeutic potential and has many intracellular targets, including nucleic acid, apoptosis, and cell cycle. Tuli et al. researched the variety of molecular mechanisms that mediate the pharmacological effects of cordycepin. Besides, they deem that cordycepin can participate in various molecular processes in cells because of its similarity with adenosine [7]. Wang et al. investigated the effects of cordycepin in prevention of focal cerebral ischemic/reperfusion (IR) injury and suggested that cordycepin has a neuroprotective effect in the ischemic brain, which is due to the inhibition of inflammation and increase of antioxidants activity related to lesion pathogenesis [23, 24]. So cordycepin could be an attractive therapeutic candidate with oral activity against I/R-associated heart diseases such as myocardial infarction [25]. Besides, cordycepin showed the obvious analgesic effect through acetic acid-induced abdominal constrictions, hot-plate test, and neurolysin inhibition assay in mice [26]. Qian et al. considered that cordycepin is a potent anti-inflammatory and analgesic medicine. There are several studies demonstrating that* C. sinensis* stimulates steroidogenesis in primary mouse Leydig cell and activates apoptosis in MA-10 mouse Leydig tumor cells in dose- and time-dependent manners [27, 28]. The steroidogenic and apoptotic mechanism of cordycepin is also clear—cordycepin stimulated intracellular PLC/PKC and MAPK signal transduction pathways to induce steroidogenesis and cell death in MA-10 mouse Leydig tumor cells [29]. In addition, cordycepin stimulated the release of some cytokines of resting PBMCs and influenced proliferation of PBMCs and transcription factors in THP-1 cell line. Accordingly, cordycepin can intensively regulate the functions of human immune cells in vitro [30]. Besides, cordycepin is a broad spectrum biocidal compound possessing not only antitumor activity but also antibacteria, antivirus, and insecticidal activities [2]. To sum up, cordycepin was confirmed as a marker for* C. militaris* within the content profiles of nucleosides in* Cordyceps* product [1].

#### 2.1.2. Adenosine

**Figure 2:**
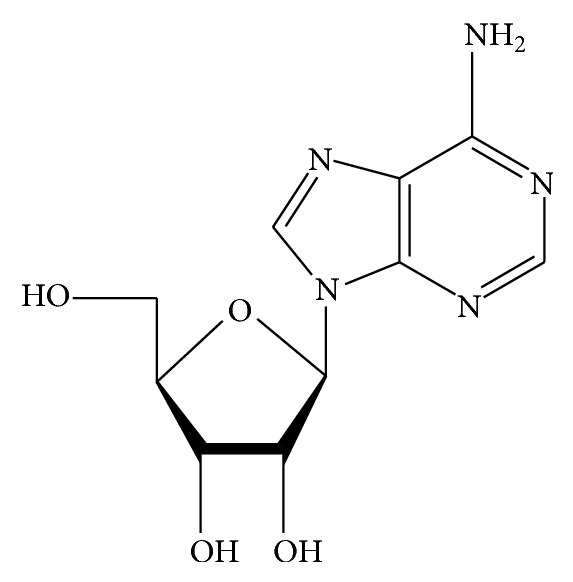
Chemical structure of adenosine.

Adenosine (Figure 2), which plays an important role in biochemical process in the organism, is a major nucleoside in* Cordyceps* spp. [31]. The content of adenosine is much higher in cultured* Cordyceps sinensis* than in the natural one. Among them, cultured* C. sinensis* has a large number of adenosines, which are much higher than those in cultured* C. militaris* [20, 31]. Nucleotide named AMP can be degraded to adenosine and the source of inosine in natural* C. sinensis* may be the oxidative deamination of adenosine [17]. Many other adenosine analogues such as 2′-deoxyadenosine, 2′3′-dideoxyadenosine, cordycepin triphosphate, and 3′-amino-3′-deoxyadenosine have also been found in* Cordyceps sinensis* [1]. Yang and Li introduced three methods to extract adenosine: organic solvent pressurized liquid extraction, boiling water extraction, and ambient temperature water extraction. They found that the extraction ratio of adenosine is much affected by extracting time and natural* Cordyceps sinensis* may contain some enzymes which can decompose adenosine [32–34].

Also, adenosine is an energy transfer and signal transductant in cells and can still exert a wide spectrum of cytoprotection or prevent tissue damage such as treating chronic heart failure, anti-inflammatory properties, and anticonvulsant activity [35–38]. In addition, adenosine is reported to suppress cell growth via diverse extrinsic and intrinsic signaling pathways. In both pathways, adenosine activates caspases in a mitochondria-dependent and/or -independent manner [39–41]. For example, Ma et al. first observed that adenosine increases ROS production in tumor cells and identified the positive feedback loop for ROS-mediated mitochondrial membrane dysfunction which amplifies the death signals in the cells [42]. However, Iannone et al. support the hypothesis that inhibition of adenosine production in tumors or inhibition of A2aR is a promising strategy to increase the effectiveness of melanoma immunotherapy, because they have done a lot of experiments proving that adenosine can limit the therapeutic effectiveness of anti-CTLA4 mAb in a mouse melanoma model [43]. In fact, adenosine mediates its effects through activation of a family of four G-protein coupled receptors, namely A_1_, A_2A_, A_2B_, and A_3_ [44]. This nucleoside plays an important role in immunity and inflammation, and the adenosine A_2A_ plays an important role in depression, locomotion, and anxiety [45]. In particular in skin cells this endogenous nucleoside, acting at one or more of its receptors, could participate in dermal tissue protection and repair. To sum up, adenosine and its analogues have received so much attention due to their various pharmacological effects.

#### 2.1.3. Nucleobases

**Figure 3:**
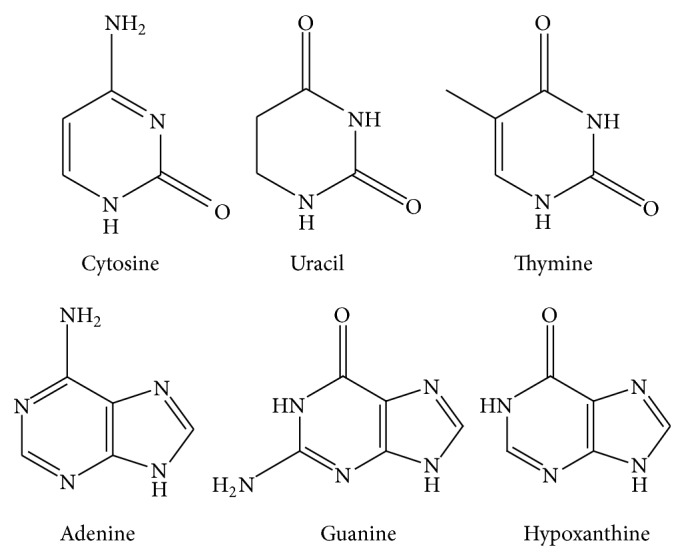
Chemical structure of six nucleosides.

To date, six nucleobases (Figure 3), including cytosine, uracil, thymine, adenine, guanine, and hypoxanthine, were determined in natural and cultured* Cordyceps sinensis*. The overall content of nucleosides is much higher in cultured* Cordyceps sinensis* than in natural ones by comparison [32]. A method based on optimum acid hydrolysis followed by high-performance liquid chromatography (HPLC) with diode array detection was developed for quantitative determination of these bioavailable nucleosides by Fan et al. which is now the most recognized appraisal method [46]. As a result, the total purine and pyrimidine bases may be the reasonable marker for evaluation of the nutrition of the materials containing nucleosides [47]. However, the pharmacological effects on nucleobases alone have not been reported currently.

#### 2.1.4. Nucleotides

Three nucleotides, namely, uridine-5′-monophosphate (UMP), adenosine-5′-monophosphate (AMP), and guanosine-5′-monophosphate (GMP), were separated by ion-pairing reversed-phase liquid chromatography-mass spectrometry (IP-RP-LC-MS) developed by Yang et al. [17]. In the pharmacological aspect, nucleotides were reported to enhance the immune response, influence metabolism of fatty acids, help the absorption of iron gut, and improve the gastrointestinal injury after repair [17, 48]. The nucleotides such as AMP, GMP, and UMP can be degraded to adenosine, guanosine, and uridine, respectively. Actually, nucleotides could be considered as an amphoteric molecule with base and phosphoric acid. Guanosine has the highest content of all in natural and artificial* Cordyceps sinensis* showed by many investigations [48]. Nucleotides can inhibit urethral inflammation, promote blood circulation, and improve brain function, and their most important effect is enhancing human immunity, which has been reported in previous studies [49].

### 2.2. Polysaccharides

*Cordyceps sinensis* contains a great deal of polysaccharides, which can be in the range of 3–8% of the total weight [50, 51].* Cordyceps* polysaccharides mainly include extracellular polysaccharide and intracellular polysaccharide. A large amount of experimental evidence has shown that fungal polysaccharides have a wide range of bioactivities including antitumor [52], anti-influenza virus [53], immunopotentiation [54], hypoglycemic [55], hypocholesterolemic [56], and antioxidant effects [51]. Other studies have suggested that the pharmacological activity of the polysaccharide was correlated with its characteristics. For example, it is polysaccharides' high molecular weight that determines the antitumor activity [57]. In other words, Sasaki et al. confirmed that the fungi polysaccharide's antitumor activity is related to the molecular weight, and the fungi polysaccharide has antitumor activity if its molecular weight is greater than 16000. But beyond that, ten monosaccharides, namely, rhamnose, ribose, arabinose, xylose, mannose, glucose, galactose, mannitol, fructose, and sorbose in 13 samples of natural and cultured* C. sinensis*, were qualitatively and quantitatively analyzed [58]. However,* Cordyceps* polysaccharides were usually composed of these monosaccharides and they play a prominent role in the organism. Polysaccharides are the main contributor towards the pharmacological properties of* C. sinensis*. Nevertheless, its application has been limited so far because of its limited supply. It is an endangered species due to the excessive harvest of the natural fungus [59, 60]. Meanwhile, cultured* C. sinensis* has shown as many pharmacological properties as natural* C. sinensis* [50].

#### 2.2.1. EPSF

Exopolysaccharide fraction (EPSF), a heteropolysaccharide, was extracted from the cultured supernatant of* C. sinensis*. The cultured supernatant was collected and then treated with three times volume of 95% ethanol for precipitation. As a result, the sediment contains a large number of EPSF [60]. EPSF has a large number of pharmacological effects; two of the most important are immunomodulatory and antitumour effects [61]. Previous reports have shown that EPSF could scavenge free radical, induce differentiation of cancer cells, and enhance antitumor ability via activating different immune responses in the host [61]. Thus it can be seen that elevating immunity is much helpful in tumor therapy. In order to explore the effects of exopolysaccharide fraction (EPSF), ICR mice were treated with EPSF for 7 days at different doses after H_22_ tumor cells' injection. The data of these studies show that EPSF could elevate the immunocytes' activities in H_22_ tumor-bearing mice, which might be closely related to elevating peritoneal macrophages' and splenic lymphocytes' activity [60]. Studies have shown that mature DCs (dendritic cells) are important modulators of immune response and their ability to initiate cytotoxic T lymphocyte is very valuable in cancer immunotherapy, and maturation of DCs is a critical factor for the initiation of immune response [62]. Song et al. found that EPSF can promote DC's maturation and activation, which is probably related to the inhibition of STAT3 phosphorylation [63]. This is another mechanism of EPSF's antitumor effect. Yang et al. have investigated the effects of the EPSF on c-Myc, c-Fos, and vascular endothelial growth factor (VEGF) expression of tumor-bearing mice using Simple PCI image analysis software. The c-Myc, c-Fos, and VEGF levels in the lungs and livers of EPSF-treated mice were found to be significantly lower than those of untreated mice, which suggests that EPSF had inhibited tumor growth in the lungs and livers of mice. As a result, it might be a potential adjuvant in cancer therapy [64]. As mentioned above, the EPSF can inhibit a variety of cancer cells; moreover, it may enhance the antitumour ability of animals or humans by activating different immune responses in the host. EPS-1, which is an exopolysaccharide produced by the medicinal fungus* Cordyceps sinensis*, has been specifically named and widely concerned. A recent study has shown that the sulfated EPS-1 derivatives have remarkable antioxidant activities. So, sulfation was an effective and favorable strategy for improving the physicochemical properties and bioactivities of fungal polysaccharides [65], which is a good idea for the study of polysaccharides.

#### 2.2.2. APS

An acid polysaccharide (APS) was isolated from cultivated* C. sinensis* mycelia by ion-exchange and sizing chromatography. APS is composed of mannose, glucose, and galactose in an approximate molar ratio of 3.3 : 2.3 : 1 [66]. In the present study, pretreatment of PC_12_ cells with APS could reduce H_2_O_2_-induced cell death, which was investigated by measuring cell viability, lactate dehydrogenase (LDH) release, antioxidant enzyme activity, malondialdehyde (MDA) levels, and intracellular accumulation of reactive oxygen species (ROS) and Ca^2+^ [66]. In conclusion, APS possesses protective effects in PC_12_ cells against H_2_O_2_-induced injury [67]. However, the antioxidant mechanism of APS remains unclear and needs further investigation. In view of the fact that acid polysaccharide fraction (APSF), extracted from* C. sinensis* fungus, has stimulating effects on macrophages [68], Chen et al. have proved that APSF may convert M_2_ macrophages to M_1_ phenotype by activating NF-*κ*B pathway. So APSF also has immunomodulatory effects as many other polysaccharides [69].

#### 2.2.3. CPS-1

A water-soluble polysaccharide named CPS-1 had been isolated from* C. sinensis* mycelium by hot water extraction, ethanol precipitation, anion-exchange, and gel permeation chromatography [70]. CPS-1 was a glucomannogalactan with the monosaccharide composition of glucose : mannose : galactose = 2.8 : 2.9 : 1. (1 →) and (1 → 3,6) linkage of glucose composed the backbone of CPS-1. Present studies have demonstrated that CSP-1 had strong antioxidation activities, which can be used to reduce the blood glucose level [71] and treat renal failure [70]. On one hand, CPS-1 can scavenge hydroxyl radicals and reduce power- and Fe^2+^-chelating. That indicated a connection between antioxidant activity and reparation of renal failure. On the other hand, CPS-1 stimulates pancreatic release of insulin and/or reduces insulin metabolism, so the polysaccharide can treat diabetes. Especially, the reducing power of CPS-1 was very potent and nearly as effective as ascorbic acid [71].

#### 2.2.4. CPS-2

**Figure 4:**
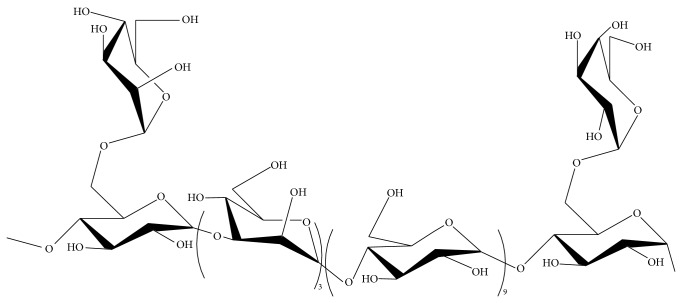
Predicted structure of CPS-2 isolated from the fruiting bodies of cultured* Cordyceps sinensis*.

CPS-2, a* Cordyceps sinensis* polysaccharide, was found to be mostly of *α*-(1 → 4)-D-glucose and *α*-(1 → 3)-D-mannose, branched with *α*-(1 → 4,6)-D-glucose every twelve residues on average (Figure 4). A monosaccharide analysis conducted by the PMP precolumn derivation method showed that CPS-2 was composed of mannose, glucose, and galactose with the ratio of 4 : 11 : 1 [72]. CPS-2, which appeared as white powder, has been demonstrated to have significant therapeutic activity against chronic renal failure. Recently, the underlying molecular mechanism has been explored by scientists. Wang et al. found that CPS-2 could reduce PDGF-BB-induced cell proliferation through the PDGF/ERK and TGF-b1/Smad pathways [73]. As a result, CPS-2 inhibits PDGF-BB-induced human mesangial cells (HMCs) proliferation in a dose-dependent manner.

#### 2.2.5. Other Polysaccharides

A neutral mannoglucan with a molecular weight of 7.7 × 10^3^ Da was obtained from the 0.05 M acetate buffer extract of* C. sinensis* mycelium. It is a branched polysaccharide with a backbone composed mainly of (1→4)- and (1→3)-linked D-glucosyl residues. Moreover, mannoglucan showed weak cytotoxicity activity against SPC-I cancer line and no obvious cytotoxicity activities against BCAP37 and SW480 cancer line [74]. Similarly, a water-soluble polysaccharide fraction, CME-1, with a molecular mass of 27.6 kDa, was prepared from* Cordyceps sinensis* mycelia and identified by NMR and GC-MS [75]. Wang et al. finally found that CME-1 can protect RAW264.7 cells against oxidative stress through inhibition of SMase activity and reduction of C_16_- and C_18_-ceramide levels [75]. In addition, a new component named cordyglucans was released by successive extractions with hot water and 0.05 M sodium hydroxide solutions. Cordyglucans were found to exhibit potent antitumor activity, which could be correlated to their (1→3)-*β*-D-glucan linkages [76]. Besides, two other polysaccharides, named CS-F10 and cordysinocan, were extracted from the cultured mycelium of* Cordyceps sinensis*, respectively. The former has the hypoglycemic activity, which can lower the plasma glucose level and decrease protein content of facilitative glucose transporter isoform 2 from rat liver following i.p. administration [55] and the latter can not only induce the cell proliferation, but also increase the phagocytosis activity and the enzymatic activity of acid phosphatase [77].

### 2.3. Sterols

Sterols components of fungus have important physiological function; they also have a variety of biological activities at the same time. So studying sterols has important theoretical significance and application prospects.

#### 2.3.1. Ergosterol

**Figure 5:**
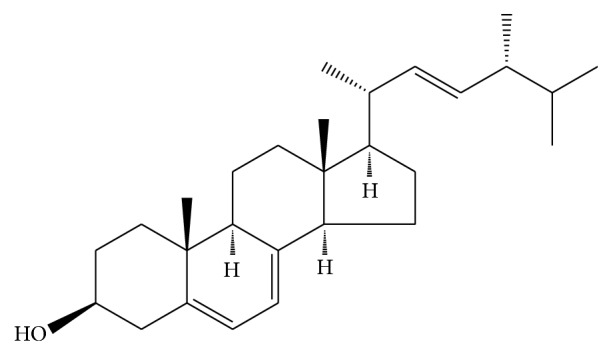
The structure of ergosterol.

Ergosterol (Figure 5) is a characteristic of fungi sterol and an important source of vitamin D_2_ [78]. Ergosterol did not get enough attention in the study of* C. sinensis* although it is a characteristic of fungi sterol [79]. Y. H. Li and X. L. Li determined the content of ergosterol in* Cordyceps sinensis* with HPLC method and obtained a high yield. They also presented that ergosterol existed in free and combined states [80]. It is important that ergosterol is a food, feed, and pharmaceutical raw material. In addition, it is an important raw material in the production of steroid hormone drugs [81]. Zheng et al. have proved the cytotoxicity and antimicrobial activity of ergosterol; it possesses weak cytotoxicity against HL-60 and BEL-7402 cell lines and moderate antimicrobial activity against the bacteria* E. aerogenes* and* P. aeruginosa* and the fungus* C. albicans* [82]. At present, the ergosterol biosynthesis pathway research has made great progress which will provide theoretical guidance to get high yield strains by genetic engineering [83].

#### 2.3.2. H1-A

**Figure 6:**
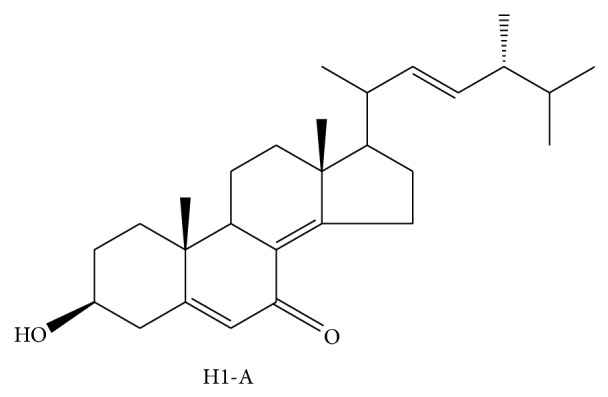
Chemical structure of compound H1-A.

H1-A (Figure 6), a pure compound used in traditional Chinese medicine, has been isolated from* C. sinensis.* To clarify the pharmacologic properties of H1-A, a series of researches studied its effect on mesangial cell proliferation, cytotoxicity, cell cycles, and apoptosis. These findings suggest that H1-A modulates some subcellular signal transduction pathways and changes the balance between proliferation and apoptosis of mesangial cells in vitro or in vivo. H1-A may be effective in the management of autoimmune disorders, and the modulation of the signal transduction proteins Bcl-2 and Bcl-XL may represent a target for future pharmacologic interventions [84]. As early as four years ago, they have reported the effect of H1-A on inhibiting autoimmune disease in MRL lpr/lpr mice. Additionally, the structure has been analyzed with NMR by Yang et al., as shown in Figure 6. It is like an ergosterol and has been proved to be without glucocorticosteroid receptor binding ability. From another point of view, H1-A is a kind of ergosterol and its structure looks like testosterone and dehydroepiandrosterone [85]. Meanwhile, H1-A can suppress the activated HMC and alleviate IgAN (Berger's disease) with clinical and histologic improvement. Lin et al. predicted that H1-A as a therapeutic regimen might be used in the future [86].

#### 2.3.3. Other Sterols

**Figure 7:**
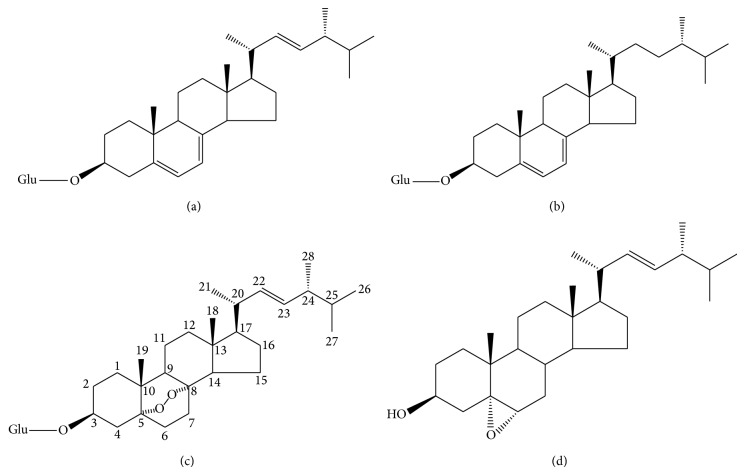
Four important sterol compounds.

Two identified compounds, ergosteryl-3-O-*β*-D-glucopyranoside (a) and 22,23-dihydroergosteryl-3-O-*β*-D-glucopyranoside (b), were isolated during the fractionation of the methanol extract of* C. sinensis* [87]. Besides, 5*α*,8*α*-epidioxy-24(R)-methylcholesta-6,22-dien-3*β*-D-glucopyranoside (c) and 5*α*,6*α*-epoxy-24(R)-methylcholesta-7,22-dien-3*β*-ol (d) are two glycoside derivatives of a sterol and they also exist in the methanol extract of* C. sinensis* [87, 88]. These are the other four important sterol compounds (Figure 7), which are sterol derivatives, and their structure as shown in Figure 7. Moreover, Matsuda et al. have confirmed through a large number of experiments that the latter two have the anticancer activity, but the first two do not [88]. The glycosylated form of ergosterol peroxide was found to be a greater inhibitor to the proliferation of K562, Jurkat, WM-1341, HL-60, and RPMI-8226 tumor cell lines [88].

### 2.4. Protein

Most of the proteins in* C. sinensis* are enzymes, including the intracellular proteases and extracellular proteases.

#### 2.4.1. CSDNase

A new acid deoxyribonuclease (DNase) that acted at an acidic pH without divalent ions was extracted from* C. sinensis* and designated CSDNase. Although acid DNase was first studied biochemically in the 1960s [89], its structure has not been elucidated. DNases may be broadly divided into two classes: DNase I and DNase II. CSDNase belongs to the latter. The protein was purified by (NH_4_)_2_SO_4_ precipitation and a series of chromatographic separations. It was found to be single-chained with an apparent molecular mass of 34 kDa and act on both dsDNA and ssDNA as a deoxyribonuclease but act preferentially on dsDNA. The activity of CSDNase was primarily expressed during fungal mycelium growth and it was an endocellular enzyme [90]. Furthermore, CSDNase was an endonuclease, which was found to hydrolyze DNA and to generate 3-phosphate and 5-OH termini. These results indicated that the nucleolytic properties of CSDNase were essentially the same as those of other well characterized acid DNases.

#### 2.4.2. CSP

A novel serine protease with fibrinolytic activity named CSP was purified from the culture supernatant of the fungus* Cordyceps sinensis*. CSP is a single polypeptide chain with an apparent molecular weight of 31 kD. It is a novel extracellular protease with a free cysteine residue near the active site. It also can hydrolyse bovine serum albumin (BSA) and human serum albumin (HSA) to a lesser extent. Li et al. have found that CSP was a plasmin-link protease but not a plasminogen activator, and it preferentially cleaved the A*α* chain of fibrinogen and the *α*-chain of fibrin. In conclusion, the presence of CSP possibly linked* C. sinensis* to its pharmacological use for cardiovascular disease, which will provide a new insight into the protein engineering of new thrombolytic agents [91].

### 2.5. Amino Acid and Polypeptide

*C. sinensis* contains many amino acids and polypeptides, which played an important role in clinical trials. For example, some polypeptide macromolecule in* C. sinensis* could significantly reduce the mean arterial pressure of rats and induce a direct endothelium-dependent vasorelaxant effect by stimulating the production of nitric oxide and endothelium-derived hyperpolarizing factor [92]. Thus, it could be used for the treatment of hypertension. As a result, it is necessary to explore the pharmacological effects of amino acid and polypeptide in* C. sinensis*.

#### 2.5.1. Cordymin

Cordymin is a peptide from the medicinal mushroom* Cordyceps sinensis* with the putative beneficial effect on diabetic osteopenia in diabetic rats. The relationship between diabetes and osteoporosis is widely studied [93, 94]. However, the mechanism of cordymin for the treatment of diabetic osteopenia is complicated. To sum up, the significant effect of cordymin on diabetic osteopenia might be directly through weakening ALP and TRAP activity and mediately through recovery of *β* cells and lowering the concentration of serum glucose, which subsequently triggered a lower extent of oxidative stress in diabetic rats [95]. All those findings indicated a major breakthrough for the treatment of diabetic osteopenia using monomer composition—cordymin.

#### 2.5.2. Cordycedipeptide A

**Figure 8:**
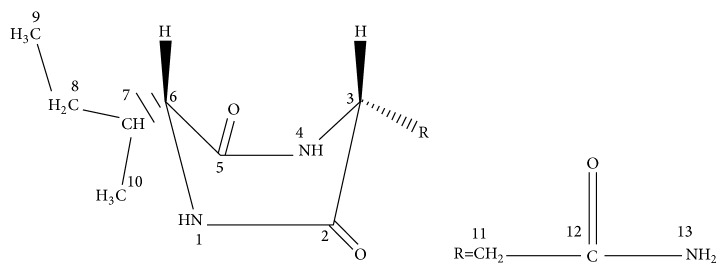
The structure of cordycedipeptide A.

A new cyclodipeptide named cordycedipeptide A was isolated from the culture liquid of* Cordyceps sinensis* (Figure 8). Its structure was elucidated as 3-acetamino-6-isobutyl-2,5-dioxopiperazine. Jia et al. have reported the cytotoxic activities of the constituent to L-929, A375, and Hela and its better effect on several tumor cell lines [96]; another pharmacological action of cordycedipeptide A remains to be further researched however.

#### 2.5.3. Cordyceamides A and B

**Figure 9:**
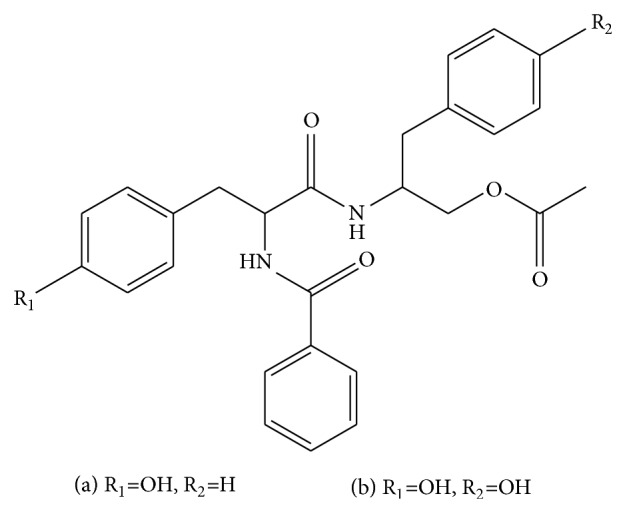
The structure of (a) and (b). (a) Cordyceamides A. (b) Cordyceamides B.

Two new aurantiamides named as cordyceamides A and B were isolated from the culture liquid of* Cordyceps sinensis* (Figure 9). Their structures were elucidated as N-benzoyl-L-tyrosinyl-L-phenylalaninol acetate and N-benzoyl-L-tyrosinyl-L-p-hydroxyphenylalaninol acetate by NMR techniques. Previous studies suggested that both Cordyceamides A and B had cytotoxic effects on L929, A375 and Hela cell lines. A showed better effect than B on L929 cell and A375 cell, but on Hela cell B showed better effect [97].

#### 2.5.4. Tryptophan

There are 18 kinds of amino acids in* C. sinensis* and they mainly played a sedative hypnotic effect. Tryptophan is the most effective ingredient among them. Tryptophan is the precursor of serotonin material, which has close relationship with animals' insomnia [98]. Otherwise, glutamic acid has the effect of immune inhibition. Due to the performance of the combined effect being more complex, more research needs to be explored.

### 2.6. Others

*C. sinensis* contains a lot of D-mannitol, also known as cordycepic acid, and the content in insect body is higher than that in stroma. Its structure has been determined as 1,3,4,5-tetrahydroxy-cyclohexanoic acid, isomeric with quinic acid. It differs mainly from the natural quinic acid in being dextrorotatory and not forming the lactone [99]. Cordycepic acid played a significant role in treating liver fibrosis of hepatic stellate cells. Liver fibrosis is a variety of factors involved in complex process [100]. Cordycepic acid ameliorates the LPs-induced inflammatory phenotype and TGF*β*1-induced fibrogenic response of cultured HSCs, which are the drug's therapeutic mechanisms to inhibit and resolve liver fibrosis [101]. Additionally, cordycepic acid in* C. sinensis* has effects on diuretic, improving the plasma osmotic pressure and anti-free radical according to pharmacological research and clinical reports [102, 103]. So it is regarded as one of the active ingredients in* C. sinensis*.

A new monosaccharide saponin, whose structure is 3-O-glucopyranoside, was isolated and identified from the mycelia of* C. sinensis*. It displayed very good antitumor activity, but the content of the mycelia was very low [104].

Moreover, there are many other ingredients in the fungi* C. sinensis*, for example, alkane, polyamine, vitamin, and microelement. Each of these has its own pharmacological effects. More constituents need to be separated and studied in the future research work, however.

## 3. Conclusion

*C. sinensis*, a macro fungus of biomedical importance, contains a number of bioactive components (Table 1). Many of them are biological response modifiers which activate our immune systems for a multitude of defensive functions. The immunomodulating effects are associated with its antitumour activity, which is the most proverbial effect of* C. sinensis*. Many ingredients in* C. sinensis* have the antitumour activity as shown above, such as cordycepin, adenosine, EPSF, cordyglucans, and monosaccharide saponins. As investigation into this fungus continues, more bioactive constituents with potential therapeutic value will be isolated. However, new methods and technologies need to be adopted to extract and analyse the components, requiring evaluation along the modern scientific line. Overall, so far, we know only a little of the wonders of this creature and it still has many secrets for us to discover. More research is needed on the herbal medicine and its related species.

## Figures and Tables

**Table 1:** Chemical constituents and their corresponding pharmacological actions of *C. sinensis*.

Chemical constituents of *C. sinensis *	Pharmacological effects	References
Cordycepin	Anti-inflammatory effect	[17, 21–30]
	Analgesic effect	
	Stimulates steroidogenesis	
	Enhances immunity	
	Antitumor activity	
	Antibacteria, antivirus, and insecticidal activities	

Adenosine	Anticonvulsant activity	[20, 31–46]
	Inhibits cancer cell growth	
	Anti-inflammatory effect	

EPSF	Immunomodulatory effect	[60–65]
	Antitumour effect	
	Antioxidant effect	

APS	Antioxidant effect	[66–69]
	Immunomodulatory effects	

CPS-1	Antioxidant effect	[70, 71]

CPS-2	Inhibits cell proliferation	[72, 73]

Mannoglucan	Cytotoxicity activity	[74]

CME-1	Antioxidant effect	[75]

Cordyglucans	Antitumour effect	[76]

CS-F10	Hypoglycemic activity	[55, 77]

Cordysinocan	Induces cell proliferation	[77]

Ergosterol	Cytotoxicity	[78–83]
	Antimicrobial activity	

H1-A	Immunoregulation	[84–86]

CSDNase	Hydrolyzes DNA	[89, 90]
	Nucleolytic properties	

CSP	Fibrinolytic activity	[91]

Cordymin	Antidiabetic	[93–95]

Tryptophan	Sedative hypnotic effect	[98]

Cordycepic acid	Treating liver fibrosis diuretic	[99–103]
	Improving the plasma osmotic pressure	
	Anti-free radical	

Monosaccharide saponins	Antitumor activity	[104]
